# BME Frontiers: A Platform for Engineering the Future of Biomedicine

**DOI:** 10.34133/2020/2095460

**Published:** 2020-04-28

**Authors:** Xingde Li, Guoqi Zhang, Yuguo Tang

**Affiliations:** ^1^Department of Biomedical Engineering, Johns Hopkins University, USA; ^2^Department of Precision and Microsystem Engineering, Delft University of Technology, Netherlands; ^3^The Suzhou Institute of Biomedical Engineering and Technology, Chinese Academy of Sciences, China

With immense enthusiasm, we introduce *Biomedical Engineering (BME) Frontiers*, the third addition to the family of Science Partner Journals. The launch of *BME Frontiers* follows a year of intense preparation. As an open-access, peer-reviewed academic journal, *BME Frontiers* covers basic research in relevant fields, as well as preclinical and clinical investigations. The content will include innovative fundamental and translational concepts, mechanisms, materials, devices, systems, processes, and methods. *BME Frontiers* will also serve as a platform for catalyzing forward-looking discussions about the field and will include perspectives on training and educating the future BME workforce.

Over the course of history, human beings have consistently sought tools and pursued knowledge to fight for wellness. The use of a sharpened stone to relieve pain, a primitive form of acupuncture in ancient China, has been traced to the Neolithic Age [1]. False toes made of wood and leather, an early form of prosthetics, were found in burial sites of aristocrats in Egypt, dating back more than 3,000 years [2]. The ancient Greek physician, Hippocrates, brought scientific spirit into medicine, using diagnostic observation and applying rational treatment instead of superstition [3].

Modern medicine would not exist without the integration of the physical sciences, biology, and engineering. As a field established more than a half century ago, BME applies electrical, chemical, optical, mechanical, data science, and other engineering principles and instruments to understand, modify, and control biological systems. BME has increasingly contributed to medicine and translational research, and its imprint can be easily seen in many recent advances in understanding human biology and improving human health. Examples include developments directly related to clinical practice, such as the creation of the cardiac pacemaker and surgical robotics. Other innovations have benefited research (as well as clinical practice), such as advanced gene and RNA-sequencing machines, mass spectrometers, and the computational tools to generate and analyze large omic datasets, 3D printing for tissue and organ regeneration, lab or organ on chip, and advanced imaging tools for biomedical research and clinical practice. BME has also been instrumental in the development of artificial intelligence for automated data mining.

BME serves as a two-way bridge between engineering and medicine. In addition to providing tools for new applications in medicine, BME also responds to the needs of and feedback from the medical community. This input from the clinical side inspires BME innovation to advance our understanding of the pathogeneses of diseases, enhance treatment options and outcomes, and ultimately improve health care and human quality of life by promoting precision and preventive care. Combining knowledge in all of the biological, medical, and engineering fields to inventively tackle some of the most difficult challenges forms the core spirit of BME.

To foster the interconnection among these multidisciplinary areas, a comprehensive publication platform for sharing and disseminating the latest BME research is needed. Many excellent journals focus on one particular BME topic, yet very few cover its broader aspects. To fulfill this unmet need, we now launch *BME Frontiers* to serve as a platform that reaches across individual fields and inspires novel discoveries and technologies and their clinical translation, as well as facilitates collaboration among the BME disciplines, to promote a brighter future for human health.

We are humbled to serve as the founding Editors-in-Chief and are grateful to the Editorial Board, which is composed of highly qualified, energetic scientists from around the world in biological, medical, and engineering fields. These experts span the multiple disciplines needed to evaluate research from different angles. Through their joint efforts and your contributions, *BME Frontiers* will become a leading must-read journal for the global BME community.
